# The influence of glatiramer acetate on Th17-immune response in multiple sclerosis

**DOI:** 10.1371/journal.pone.0240305

**Published:** 2020-10-30

**Authors:** Mikhail Melnikov, Svetlana Sharanova, Anastasiya Sviridova, Vladimir Rogovskii, Nina Murugina, Anna Nikolaeva, Yulia Dagil, Vladimir Murugin, Tatiana Ospelnikova, Alexey Boyko, Mikhail Pashenkov

**Affiliations:** 1 Department of Neurology, Neurosurgery and Medical Genetics, Pirogov Russian National Research Medical University, Moscow, Russia; 2 Laboratory of Clinical Immunology, National Research Center Institute of Immunology of the Federal Medical-Biological Agency of Russia, Moscow, Russia; 3 Department of Neuroimmunology, Federal Center of Brain Research and Neurotechnology of the Federal Medical-Biological Agency of Russia, Moscow, Russia; 4 Department of Molecular Pharmacology and Radiobiology, Pirogov Russian National Research Medical University, Moscow, Russia; 5 Laboratory of Interferons, I.I. Mechnikov Research Institute of Vaccines and Sera, Moscow, Russia; Institut National de la Santeet de la Recherche Medicale (INSERM), FRANCE

## Abstract

Glatiramer acetate (GA) is approved for the treatment of multiple sclerosis (MS). However, the mechanism of action of GA in MS is still unclear. In particular, it is not known whether GA can modulate the pro-inflammatory Th17-type immune response in MS. We investigated the effects of original GA (Copaxone^®^, Teva, Israel) and generic GA (Timexone^®^, Biocad, Russia) on Th17- and Th1-type cytokine production *in vitro* in 25 patients with relapsing-remitting MS and 25 healthy subjects. Both original and generic GA at concentrations 50–200 μg/ml dose-dependently inhibited interleukin-17 and interferon-γ production by anti-CD3/anti-CD28-activated peripheral blood mononuclear cells from MS patients and healthy subjects. This effect of GA was reproduced using purified CD4^+^ T cells, suggesting that GA can directly modulate the functions of Th17 and Th1 cells. At high concentrations (100–200 μg/ml), GA also suppressed the production of Th17-differentiation cytokines (interleukin-1β and interleukin-6) by lipopolysaccharide (LPS)-activated dendritic cells (DCs). These GA/LPS-treated DCs induced lower interleukin-17 and interferon-γ production by autologous CD4^+^ T cells compared to LPS-treated DCs. These data suggest that GA can inhibit Th17-immune response and that this inhibitory effect is preferentially exercised by direct influence of GA on T cells. We also demonstrate a comparable ability of original and generic GA to modulate pro-inflammatory cytokine production.

## Introduction

Multiple sclerosis (MS) is a chronic inflammatory demyelinating and neurodegenerative disease of the central nervous system (CNS) with the autoimmune mechanism of development.

Glatiramer acetate (GA) (previously known as copolymer 1) is approved for the treatment of relapsing forms of MS as a first-line therapy [1]. Glatiramer acetate is a random synthetic copolymer of four amino acids (L-glutamine, L-lysine, L-alanine, L-tyrosine (GLAT)) that are most common in myelin basic protein [2]. Despite the fact that the effect of GA on experimental autoimmune encephalomyelitis (EAE) and MS pathogenesis has been under investigation for more than twenty years, the mechanism of action of GA is still not fully understood. It is assumed that GA may modulate functions of both the innate and adaptive immune systems. It was reported that GA may induce the shift of the T cell balance from a dominant pro-inflammatory phenotype (Th1) to an anti-inflammatory phenotype (Th2 / Foxp3 T regulatory cells (Treg), as well as increase the production of anti-inflammatory cytokines such as interleukin-10 (IL-10) and transforming growth factor β (TGF-β)) [3–5].

It has also been shown that GA can influence the function of B cells which may modulate inflammation in CNS by producing antibodies, pro-inflammatory cytokines and presenting antigen to effector T cells [6]. Glatiramer acetate modulates B cells functions by converting memory B cells known to contribute to the T cell-dependent inflammatory response into B regulatory cells and reduce the production of the pro-inflammatory cytokines IL-6 and tumor necrosis alpha factor (TNF-α) by B cells [7]. B cells obtained from MS patients treated with GA failed to proliferate in response to high-dose CD40 ligand when combined with additional activation stimuli [8].

There are numerous data on the influence of GA on myeloid-derived antigen-presenting cells (APCs) [9–11]. It was shown that the treatment with GA reduced the level of DCs expressing CD40 in whole blood in MS patients [9]. Monocytes obtained from GA-treated MS patients secrete high amounts of the anti-inflammatory cytokine IL-10 and less pro-inflammatory cytokine IL-12 [10]. It has also been shown that GA reduces the *in vitro* number of mature DCs expressing CD83 and HLA-DR [10]. According to some authors, APCs may be the initial cellular targets for GA and play central role in mechanism of action of GA [12].

Recent studies have demonstrated the potential importance of Th17 cells in EAE and MS pathogenesis [13, 14]. Th17-cells produce pro-inflammatory cytokines such as IL-17 and interferon-γ (IFN-γ) [13–16]. The influence of GA on MS pathogenesis could be mediated through modulation of Th17-function. However, the effect of GA on Th17-type immune response has not been studied. The purpose of this study was to clarify the effects of GA on Th17 cells and DCs-mediated Th17-immune response in MS patients and to compare this effect between generic and original GA.

## Materials and methods

### Patients

Twenty-five patients with a documented diagnosis of MS according to McDonald criteria (modification 2010) were examined [17]. All patients had a relapsing-remitting form of the disease. Their main demographic and clinical characteristics are shown in Table 1. All patients were subjected to a standard neurological examination with assessment of the expanded disability status scale (EDSS) score [18].

**Table 1:** Clinical and demographic data of MS patients and healthy subjects. Data are medians (25^th^; 75^th^ percentiles).

Factor	MS patients, n = 25	Healthy subjects, n = 25
Age, years	27 (25; 33)	26 (24; 29)
Men/women (% women)	10/15 (60)	11/14 (56)
Duration of MS, years	4 (3.3; 4.8)	NA
Duration of therapy with GA, years	2.8 (2; 3.5)	NA
EDSS score	1.5 (1; 3)	NA

EDSS, expanded disability status scale; MS, multiple sclerosis; NA, not applicable.

All patients were examined during clinical remission. All patients had been treated with generic GA (Timexone^®^, Biocad, Russia) [19] for more than one year (20 mg of GA subcutaneously daily). At the time of blood sampling, all the patients studied had not been treated with corticosteroid therapy for more than three months. The control group consisted of 25 healthy donors matched with patients by sex and age (Table 1). All patients signed the written informed consent to participate in this study.

The study was approved by the ethics committee of the Pirogov Russian National Research Medical University (protocol №179).

### Culture and stimulation of peripheral blood mononuclear cells and CD4^+^ T cells

To determine functional activity of Th17 and Th1 cells, peripheral blood mononuclear cells (PBMCs) were isolated from whole blood by centrifugation against density gradient of ficoll-urografin (PanEco, Russia), washed three times with PBS, and resuspended in RPMI-1640 medium supplemented with 2 mM L-glutamine and 2% human AB serum (both from PAA, Austria). Then, PBMCs at a concentration of 8x10^4^ per 200 μl per well were plated in 96-well U-bottomed culture plates in duplicates and stimulated with microbeads coated with anti-CD3 and anti-CD28-antibodies (Life Technologies, Norway) for 72 hours in CO_2_-incubator [20], whereafter culture supernatants were collected and stored at –70°C. Negative control samples were cultured without stimulation.

To assess the effect of GA on the function of Th1 and Th17 cells, samples of PBMCs were incubated with original GA (Copaxone^®^, Teva, Israel) [1] or generic GA (Timexone^®^, Biocad, Russia) at concentrations of 50 μg/ml, 100 μg/ml and 200 μg/ml for 15 min [21], whereafter anti-CD3/anti-CD28 microbeads were added to the cultures and the stimulation proceeded as described above.

In some experiments, CD4^+^ T cells were isolated from PBMCs by magnetic cell sorting (MACS) using negative CD4^+^ T cell isolation kit according to manufacturer’s instructions (Miltenyi Biotec, Germany). The purity of CD4^+^ T cells was > 97%, as measured by flow cytometry. Then CD4^+^ T cells were stimulated with anti-CD3/anti-CD28 microbeads as described above.

### Culture and stimulation of human immature myeloid dendritic cells and CD4^+^ T cell stimulation by activated dendritic cells

To generate human immature myeloid dendritic cells (DCs), PBMCs were isolated from whole blood by centrifugation against density gradient of ficoll-urografin (PanEco, Russia). After three washes, PBMCs were resuspended in RPMI-1640 medium supplemented with 0.5% human AB serum (both from PAA) at 10^7^ cells per ml. Then PBMCs were plated in flat-bottomed plate (Petri dish) at 6x10^5^ cells and incubated for 1 hr at 37°C in 5% CO_2_-incubator. Non-adherent cells were removed by two washes with warm RPMI-1640 medium, and wells were filled with complete culture medium (CCM), which was RPMI supplemented with 2 mM L-Glutamine and 2% human AB serum (both from PAA, Austria).

Then monocytes were cultured for 6 days in CCM supplemented with 80 ng/ml recombinant human granulocyte-macrophage colony-stimulating factor (GM-CSF) and 50 ng/ml recombinant human IL-4 (both from Miltenyi Biotec, Germany). Medium was refreshed on day 3. By flow cytometry, the phenotype of DCs was CD11c^+^HLA-DR^+^CD14^−^. The purity of cells with regard to these phenotypes was > 85% (S1 Fig). On day 6, DCs were trypsinized, counted, and replated in 96-well plates in CCM at 4x10^4^/well and incubated in duplicates with IFN-γ (100 IU/ml) (Becton Dickinson, USA) for 2 hr at 37°C in 5% CO_2_. Then cells were stimulated with lipopolysaccharide (LPS) (0.1 μg/ml) for 24 hrs, whereafter supernatants were collected. Negative control samples were cultured without stimulation [22].

To assess the effects of GA on the function of DCs, samples of DCs were incubated in the presence of generic GA at concentrations of 50 μg/ml, 100 μg/ml and 200 μg/ml for 15 min, whereafter IFN-γ and LPS were added to the cultures as described above [23].

To stimulate T cells, DCs were co-cultured with autologous CD4^+^ T cells (8x10^4^ T cells / 4x10^4^ DCs per well) in the presence of staphylococcal enterotoxin B (100 pg/ml; Sigma-Aldrich, USA) for 72 hrs, whereafter culture supernatants were collected and stored at –70°C [24]. As a positive control, CD4^+^ T cells were stimulated with anti-CD3/anti-CD28 microbeads without DCs as described above. As negative controls, CD4^+^ T cells were cultured without DCs, SEB or microbeads, or with autologous DCs that had not been treated with LPS/IFN-γ and SEB.

### Real-time polymerase chain reaction (RT-PCR)

CD4^+^ T cells were harvested after 72 hrs of stimulation with anti-CD3/anti-CD28 microbeads in the presence or absence of GA as described above. Total RNA was extracted using Total RNA purification kit from Jena Bioscience (Germany). 0.5 μg total RNA was reverse-transcribed using Revertaid^®^ reverse transcription kit (Fermentas, Lithuania). Amplifications were done in a 7300 Real-Time PCR System (Applied Biosystems, USA) using a reagent mix containing SYBR Green (Evrogen, Russia). Sequences of primers are listed in S1 Table. Cycling conditions were as follows: 95°C (5 min), then 40 cycles of 95°C (15 sec) and 60°C (45 s, detection step). Melting curves were analysed after each amplification to confirm specificity of signal. Relative expression of mRNA was calculated by the 2^−ΔΔCt^ method using unstimulated CD4^+^ T cells from the given donor and MS patients as the reference sample and GAPDH expression for normalization [25]. Data are expressed as the percentage of mRNA expression in stimulated cells in the absence of GA.

### Cytokine evaluation

Levels of IFN-γ, IL-10, IL-1β and IL-6 in the culture supernatants were determined by ELISA (Vector-Best, Russia). Lower limit of detection were 2.0 pg/ml for IFN-γ, 1.0 pg/ml for IL-10 and IL-1β, 0.5 pg/ml for IL-6. Levels of IL-17 were determined by ELISA kits from eBioscience (USA). Lower limit of detection was 0.5 pg/ml. In all cases of ELISAs, the instructions of the kit manufacturers were followed. Data are expressed as pg/ml or as the percentage of cytokine production by stimulated cells in the absence of GA.

### Statistical analysis

The statistical analysis of the results was performed using Prizm 6 software. The nonparametric Mann-Whitney U test was used to compare two groups. Differences were considered statistically significant if p<0.05.

## Results

### The impact of GA on cytokine production by activated PBMCs from MS patients and healthy subjects

According to ELISA, the production of IL-17 and IFN-γ by unstimulated PBMCs or stimulated with anti-CD3/anti-CD28 was not different between the groups (Table 2).

**Table 2:** The secretion of IL-17 and IFN-γ by PBMCs in MS patients and in healthy subjects. Data are medians (25^th^; 75^th^ percentiles).

Cytokine	Stimulation	MS patients, n = 25	Healthy subjects, n = 25
IL-17, pg/ml	None	2 (1; 2)	2 (1; 3)
	Anti-CD3 / anti-CD28	409 (247; 756)	394 (259; 776)
IFN-γ, pg/ml	None	22 (17; 26)	13 (11; 18)
	Anti-CD3 / anti-CD28	4620 (3323; 6348)	4644 (4212; 5536)

IFN-γ, interferon-γ; IL-17, interleukin-17; MS, multiple sclerosis.

**Fig 1:**
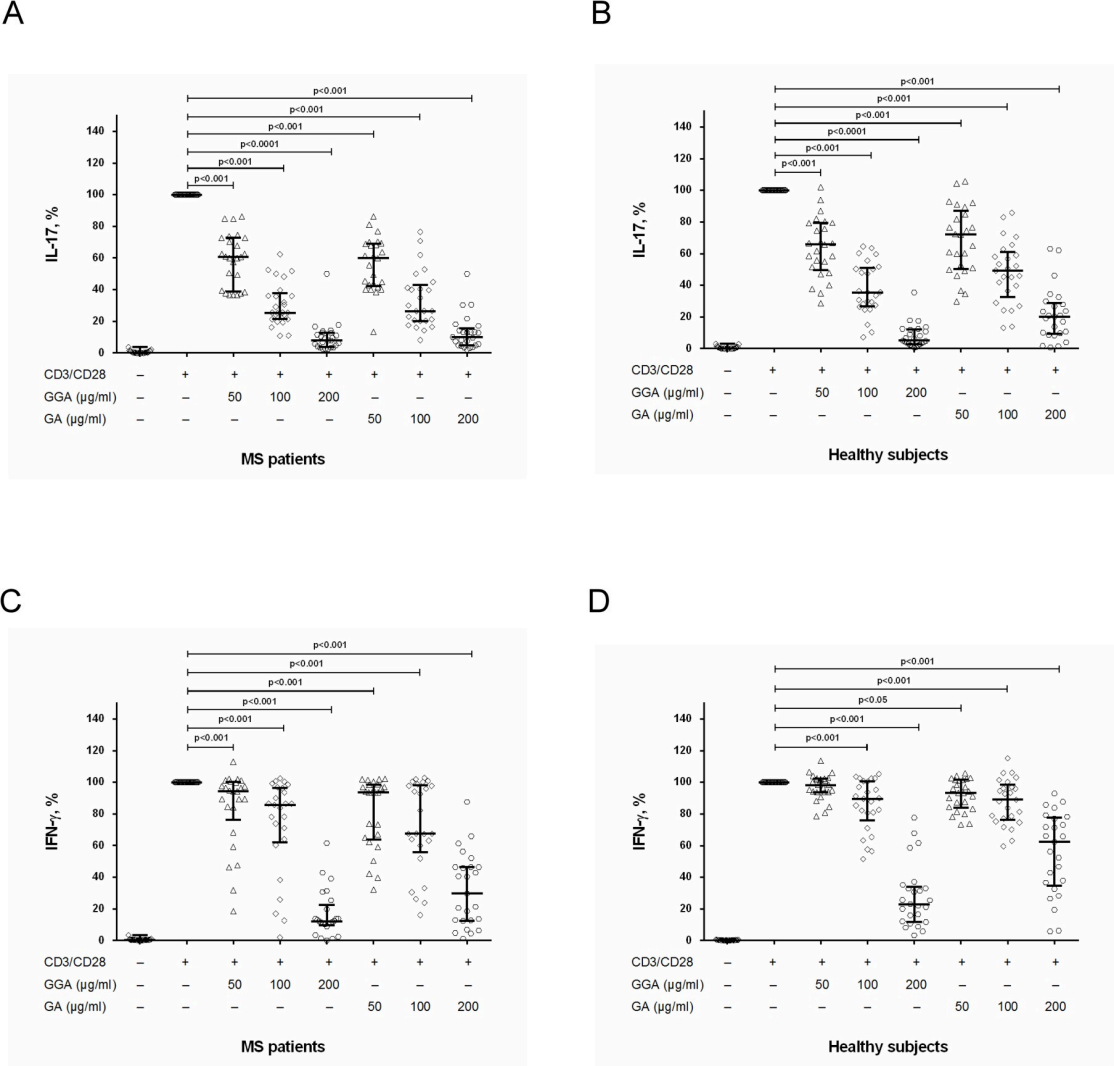
The influence of glatiramer acetate on IL-17 and IFN-γ production by activated PBMCs in MS patients and in healthy subjects. Peripheral blood mononuclear cells (PBMCs) (8 х 10^4^ per 200 μl per well) obtained from MS patients in clinical remission or from healthy subjects were activated with microbeads coated with anti-CD3 and anti-CD28 antibodies with or without generic glatiramer acetate (GGA) or original GA at concentrations of 50 μg/ml, 100 μg/ml and 200 μg/ml. After 72 hours, the supernatants were collected and submitted to IL-17 (A, B) and IFN-γ (C, D) quantification by ELISA. Horizontal lines correspond to the median; whiskers correspond to 25^th^ and 75^th^ percentiles. The median values of control and generic GA or original GA treated groups were compared and the p values are indicated at the figure.

To study possible influence of GA on production of pro-inflammatory cytokines (IL-17, IFN-γ), we stimulated PBMCs with anti-CD3/anti-CD28 in the presence of original or generic GA at concentrations of 50 μg/ml, 100 μg/ml and 200 μg/ml. We found that original and generic GA significantly and dose-dependently reduced IL-17 production by stimulated PBMCs in both groups (Fig 1A and 1B). It is important that in MS patients, the effect of original and generic GA on IL-17 production by stimulated PBMCs was comparable (Fig 1A) while in healthy subjects the inhibitory effect of generic GA (at a concentration of 200 μg/ml) on IL-17 production was significantly higher than effect of original GA (Fig 1B).

We also found that original and generic GA dose-dependently reduced IFN-γ production by stimulated PBMCs in MS patients (Fig 1C). Generally, the effect of original and generic GA on IFN-γ production by stimulated PBMCs was also comparable; however, the inhibitory effect of generic GA (at a concentration of 200 μg/ml) on this production was significantly higher than the effect of original GA (Fig 1C). A different picture was observed obtained when we studied the effect of GA on IFN-γ production in healthy subjects. Here, original GA at all concentrations significantly reduced IFN-γ production by stimulated PBMCs, while the generic GA reduced this production only at concentrations of 100 μg/ml and 200 μg/ml while at a concentration of 50 μg/ml generic GA had no effect on IFN-γ production (Fig 1D). At the same time, at a concentration of 200 μg/ml the inhibitory effect of generic GA was higher than the inhibitory effect of original GA (Fig 1D).

### The impact of GA on cytokine production by activated CD4^+^ T cells cultures from MS patients and healthy subjects

In PBMCs cultures, GA can affect cytokine production by T cells either directly or indirectly by modulating functions of antigen-presenting cells such as monocytes or DCs. First, we studied direct effects of GA on the production of IL-17, IFN-γ and IL-10 by purified CD4^+^ T cells. We found that IL-17, IFN-γ and IL-10 production by unstimulated CD4^+^ T cells or stimulated with anti-CD3/anti-CD28 are comparable between the groups (Table 3).

**Table 3:** The secretion of IL-17, IFN-γ and IL-10 by CD4^+^ T cells in MS patients and in healthy subjects. Data are medians (25^th^; 75^th^ percentiles).

Cytokine	Stimulation	MS patients, n = 16	Healthy subjects, n = 15
IL-17, pg/ml	None	4 (0; 8)	1 (0; 2)
	Anti-CD3 / anti-CD28	834 (544; 1480)	975 (674; 1490)
IFN-γ, pg/ml	None	12 (11; 18)	18 (10; 21)
	Anti-CD3 / anti-CD28	9633 (4363; 11475)	6459 (5887; 7170)
IL-10, pg/ml	None	4 (2; 6)	11 (5; 15)
	Anti-CD3 / anti-CD28	294 (139; 548)	396 (315; 1467)

IFN-γ, interferon-γ; IL-10, interleukin-10; IL-17, interleukin-17; MS, multiple sclerosis.

**Fig 2:**
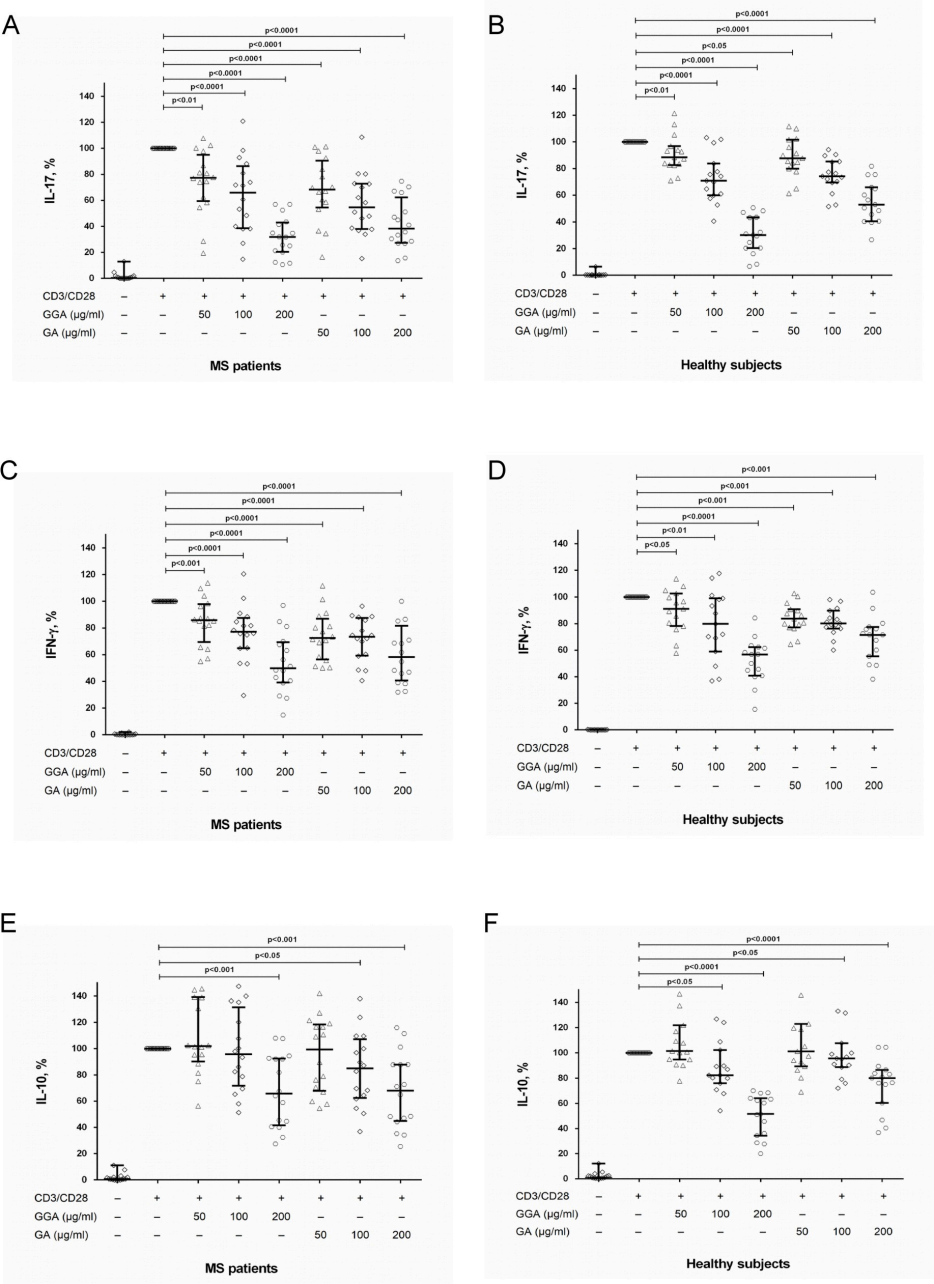
The influence of glatiramer acetate on IL-17, IFN-γ and IL-10 production by activated CD4^+^ T cells in MS patients and in healthy subjects. Data are medians (25^th^; 75^th^ percentiles). CD4^+^ T cells (8 х 10^4^ per 200 μl per well) obtained from MS patients in clinical remission or from healthy subjects were activated with microbeads coated with anti-CD3 and anti-CD28 antibodies with or without generic glatiramer acetate (GGA) or original GA at concentrations of 50 μg/ml, 100 μg/ml and 200 μg/ml. After 72 hours, the supernatants were collected and submitted to IL-17 (A, B), IFN-γ (C, D) and IL-10 (E, F) quantification by ELISA. Horizontal lines correspond to the median; whiskers correspond to 25^th^ and 75^th^ percentiles. The median values of control and generic GA or original GA treated groups were compared and the p values are indicated at the figure.

Original and generic GA dose-dependently reduced IL-17 and IFN-γ production by stimulated CD4^+^ T cells in both groups (Fig 2A–2D). The effect of original and generic GA was comparable (Fig 2A–2D). Thus, the influence of GA on IL-17 and IFN-γ production by CD4^+^ T cells corresponds with the effect of GA on PBMCs culture.

In addition, we studied the effect of GA on the production of anti-inflammatory cytokine IL-10 by CD4^+^ T cells. We found that in MS patients, original or generic GA did not affect IL-10 production at a concentration of 50 μg/ml (Fig 2E). At a concentration of 100 μg/ml, the original GA inhibited this production (Fig 2E). At a concentration of 200 μg/ml, both original and generic GA suppressed IL-10 production by activated CD4^+^ T cells (Fig 2E). In healthy subjects, original and generic GA at a concentration of 50 μg/ml did not affect IL-10 production (Fig 2F), inhibited IL-10 production at concentrations of 100 μg/ml, and 200 μg/ml (Fig 2F).

### The impact of GA on IL-17, IFN-γ and IL-10 mRNA expression in stimulated CD4^+^ T cells cultures from MS patients and healthy subjects

**Fig 3:**
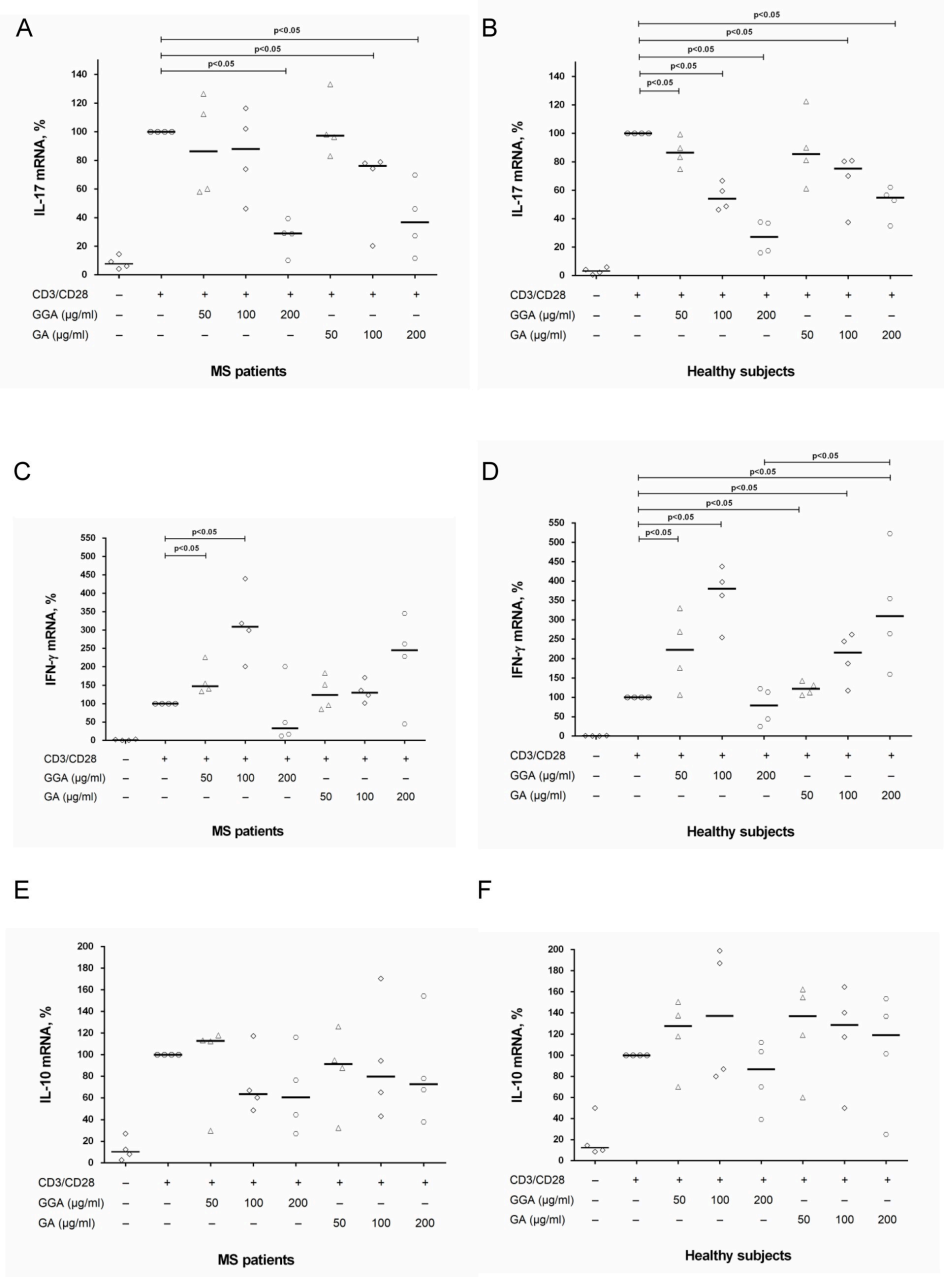
The influence of glatiramer acetate on IL-17, IFN-γ and IL-10 mRNA expression in stimulated CD4^+^ T cells in MS patients and in healthy subjects. CD4^+^ T cells (8 х 10^4^ per 200 μl per well) obtained from MS patients in clinical remission or from healthy subjects were activated with microbeads coated with anti-CD3 and anti-CD28 antibodies with or without generic glatiramer acetate (GGA) or original GA at concentrations of 50 μg/ml, 100 μg/ml and 200 μg/ml. After 72 hours, CD4^+^ T cells were harvested and total RNA was extracted. The total RNA was reverse-transcribed and submitted to relative mRNA expression of IL-17 (A, B), IFN-γ (C, D) and IL-10 (E, F) quantification by Real-Time PCR. Horizontal lines correspond to the median. The median values of control and generic GA or original GA treated groups were compared and the p values are indicated at the figure.

To clarify the effect of GA on T cells, we also investigated the expression of IL-17, IFN-γ and IL-10 mRNA in CD4^+^ T cells stimulated by anti-CD3/anti-CD28 microbeads in the presence of generic or original GA at concentrations of 50 μg/ml, 100 μg/ml and 200 μg/ml. In MS patients, generic GA suppressed IL-17 mRNA expression in CD4^+^ T cells at a concentration of 200 μg/ml, while original GA at concentrations of 100 μg/ml and 200 μg/ml (Fig 3A). In healthy subjects, generic GA dose-dependently reduced mRNA expression of IL-17 in all concentrations, while original at concentrations of 100 μg/ml and 200 μg/ml (Fig 3B). In whole, the effect of GA on mRNA expression of IL-17 corresponds with data of influence of GA on IL-17 production by activated CD4^+^ T cells. Conversely, stimulation of CD4^+^ T cells in the presence of low doses of generic or original GA (50 μg/ml and 100 μg/ml) enhanced IFN-γ mRNA expression in both groups (Fig 3C and 3D). Furthermore, in healthy subjects, original GA increased IFN-γ mRNA expression not only at concentrations of 50 μg/ml and 100 μg/ml but also at a concentration of 200 μg/ml (Fig 3D). There was no significant effect of generic or original GA on IL-10 mRNA expression in both groups (Fig 3D and 3E).

### The impact of GA on DC-mediated Th17 cell response in MS patients and healthy subjects

**Fig 4:**
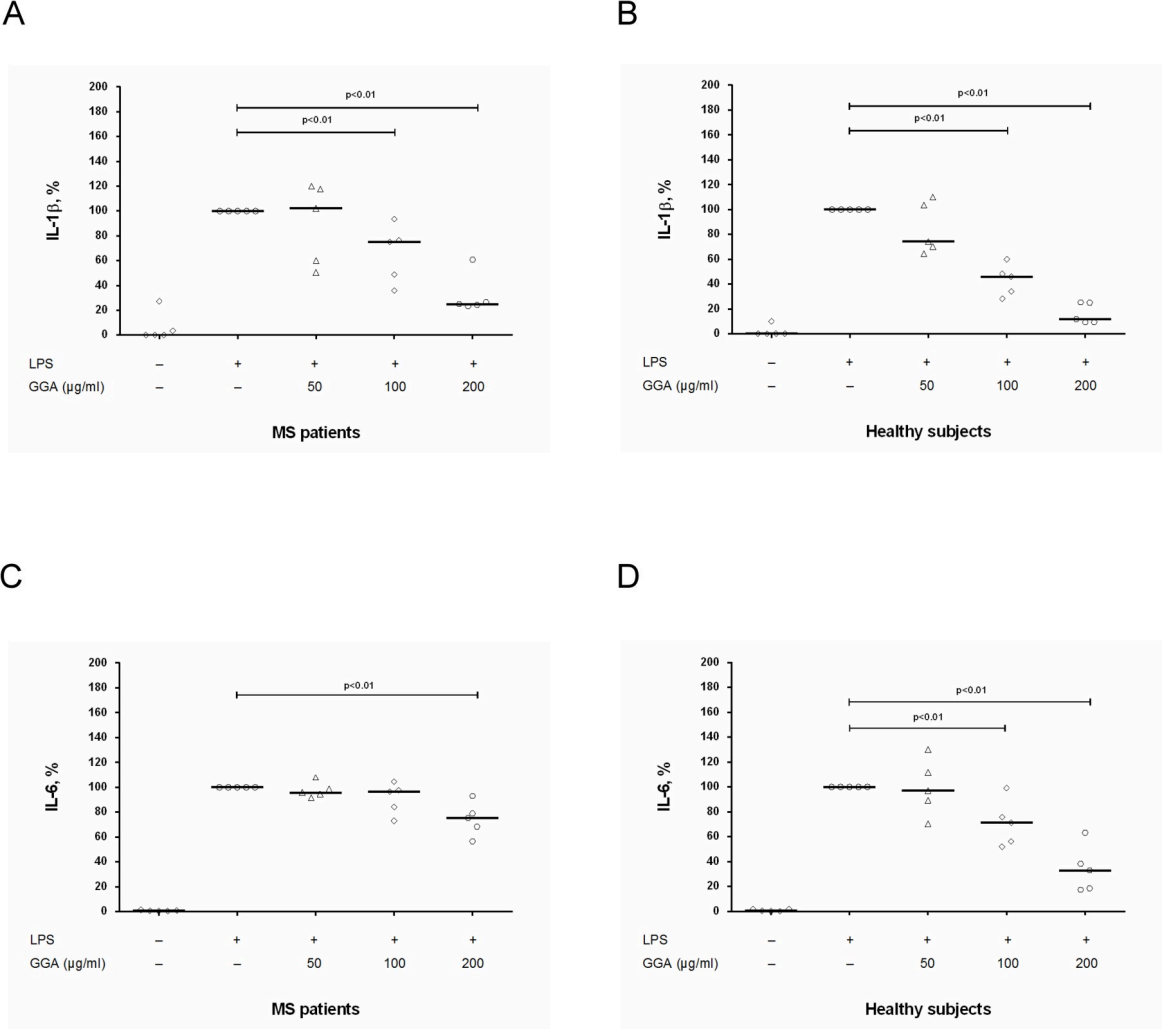
The influence of glatiramer acetate on IL-1β and IL-6 production by activated DCs in MS patients and in healthy subjects. Dendritic cells (DCs) cultures (4 х 10^4^ per 200 μl per well) obtained from MS patients in clinical remission or from healthy subjects were activated by lipopolysaccharide (LPS) with or without generic glatiramer acetate (GGA) at concentrations of 50 μg/ml, 100 μg/ml and 200 μg/ml. After 24 hours, the supernatants were collected and submitted to IL-1β (A, B) and IL-6 (C, D) quantification by ELISA. Horizontal lines correspond to the median. The median values of control and generic GA treated groups were compared and the p values are indicated at the figure.

To study the effect of GA on production of Th17-differentiation cytokines IL-6 and IL-1β by DCs, we stimulated DCs with LPS in the absence/presence of generic GA at concentrations of 50 μg/ml, 100 μg/ml and 200 μg/ml, whereafter levels of cytokines were assessed in culture supernatants by ELISA. Production of IL-1β and IL-6 by unstimulated or LPS-stimulated DCs was comparable between MS patients and healthy subjects (Table 4). At concentrations of 100 μg/ml and 200 μg/ml, generic GA significantly reduced (in dose depend manner) IL-1β production by stimulated DCs in both groups (Fig 4A and 4B). In MS patients, generic GA suppressed IL-6 production by DCs only at a concentration of 200 μg/ml (Fig 4C), while in healthy subjects at concentrations of 100 μg/ml and 200 μg/ml (Fig 4D).

**Table 4:** The secretion of IL-1β and IL-6 by DCs in MS patients and in healthy subjects. Data are medians (25^th^; 75^th^ percentiles).

Cytokine	Stimulation	MS patients, n = 5	Healthy subjects, n = 5
IL-1β, pg/ml	None	0 (0; 4)	0 (0; 0)
	LPS	111 (34; 140)	141 (85; 261)
IL-6, pg/ml	None	70 (36; 96)	11 (3; 262)
	LPS	12956 (10765; 13518)	13583 (9117; 16474)

IL-1β, interleukin-1β; IL-6, interleukin-6; LPS, lipopolysaccharide; MS, multiple sclerosis.

**Fig 5:**
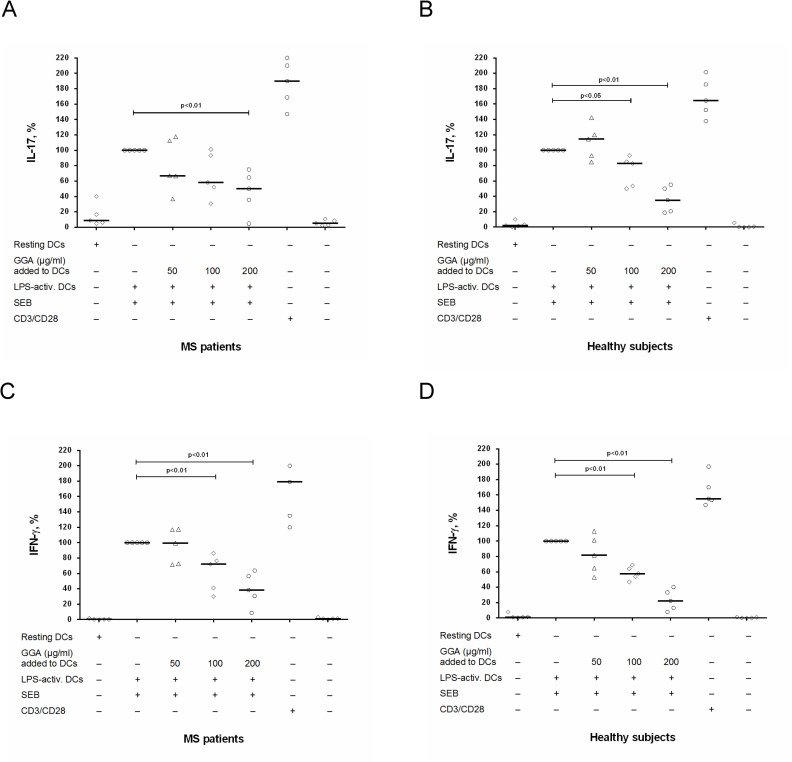
The influence of glatiramer acetate on the ability of DCs to modulate IL-17 and IFN-γ production by CD4^+^ T cells in MS patients and in healthy subjects. CD4^+^ T cells (8 х 10^4^ per 200 μl per well) obtained from MS patients in clinical remission or from healthy subjects were co-cultured with autologous dendritic cells (DCs) (4 х 10^4^ per 200 μl per well) that had been stimulated by lipopolysaccharide (LPS) in the presence / absence of generic glatiramer acetate (GGA) at concentrations of 50 μg/ml, 100 μg/ml and 200 μg/ml. CD4^+^ T cells were also stimulated with microbeads coated with anti-CD3 and CD28-antibodies (positive control) or cultured without stimuli (negative control). After 72 hours, the supernatants were collected and submitted to IL-17 (A, B) and IFN-γ (C, D) quantification by ELISA. Horizontal lines correspond to the median. The median values of control and generic GA treated groups were compared and the p values are indicated at the figure.

To assess the effect of GA on the ability of DCs to activate Th17-cells, CD4^+^ T cells were co-cultured with LPS-activated autologous DCs pretreated with different concentrations of generic GA (50 μg/ml, 100 μg/ml and 200 μg/ml), whereafter IL-17 and IFN-γ were assessed in culture supernatants by ELISA. Production of IL-17 and IFN-γ by CD4^+^ T cells activated with LPS-stimulated DCs was comparable between MS patients and healthy subjects (Table 5). We found that the treatment of DCs with generic GA at a concentration of 50 μg/ml had no effect on IL-17 and IFN-γ production by CD4^+^ T cells in both groups (Fig 5). The treatment of DCs with GA at a concentration of 100 μg/ml suppressed IL-17 and IFN-γ production in healthy subjects (Fig 5B and 5D), but had no effect in MS patients (Fig 5A and 5C). The treatment of DCs with GA at a concentration of 200 μg/ml suppressed IL-17 and IFN-γ production in both groups (Fig 5).

**Table 5:** The secretion of IL-17 and IFN-γ by CD4^+^ T cells activated with autologous DCs in MS patients and in healthy subjects. Data are medians (25^th^; 75^th^ percentiles).

Cytokine	Experimental conditions	MS patients, n = 5	Healthy subjects, n = 5
IL-17, pg/ml	Resting DCs,	28 (8; 42)	16 (2; 52)
	– SEB		
	LPS-activated DCs,	160 (115; 170)	158 (151; 232)
	+ SEB		
IFN-γ, pg/ml	Resting DCs,	6 (5; 33)	15 (7; 35)
	– SEB		
	LPS-activated DCs,	2459 (2060; 4094)	2819 (2355; 3393)
	+ SEB		

IFN-γ, interferon-γ; IL-17, interleukin-17; LPS, lipopolysaccharide; MS, multiple sclerosis; SEB, staphylococcal enterotoxin B.

## Discussion

Glatiramer acetate is one of the first therapeutics approved for treatment of relapsing-remitting MS. Although more than ten disease-modifying therapies (DMTs) with different mechanisms of action are now available for RRMS, GA has remained popular, especially considering some of the potentially life-threatening side effects of other DMTs [26].

At the same time, the mechanism of action of GA in MS is still unclear. Here, we show that the effect of GA on MS pathogenesis could be mediated by modulating the Th17-branch of immune system which plays a critical role in MS. GA dose-dependently reduced IL-17 and IFN-γ production by PBMCs and purified CD4^+^ T cells (Figs 1 and 2). Importantly, GA at concentrations of 50 μg/ml and 100 μg/ml may have a clear inhibitory effect on the production of IL-17 and IFN-γ by PBMCs and CD4^+^ T cells in MS patients (Figs 1A, 1C, 2A and 2C) without affecting cell viability and proliferative responses [21]. Conversely, there was no inhibitory effect of GA (50 μg/ml) on production of anti-inflammatory cytokine IL-10 by stimulated CD4^+^ T cells in MS patients and in healthy subjects (Fig 2E and 2F).

The influence of GA on IL-17 production by stimulated CD4^+^ T cells is in line with data on the impact of high concentrations of GA on IL-17 mRNA expression in CD4^+^ T cells (Fig 3A and 3B). It was shown that in MS patients, generic GA suppressed IL-17 mRNA expression in CD4^+^ T cells at a concentration of 200 μg/ml, while original GA at concentrations of 100 μg/ml and 200 μg/ml (Fig 3A and 3B). In healthy subjects, generic GA dose-dependently reduced mRNA expression of IL-17 in all concentrations, while original at concentrations of 100 μg/ml and 200 μg/ml (Fig 3B). At the same time at a concentration of 50 μg/ml original GA had no effect on IL-17 mRNA expression in both groups (Fig 3A and 3B) and generic GA did not affect IL-17 mRNA expression in MS patients (Fig 3A). It is important to note that the IL-17 protein level in the supernatant after 72-hours stimulation (ELISA) reflects the dynamics of mRNA expression over the entire period from 0 to 72 hours. It is possible to suggest that the effects of GA at earlier time points may differ from those observed after 72 hours. The various impact of GA on IL-17 mRNA expression in CD4^+^ T cells in MS patients and healthy subjects could be explained by the treatment with GA in MS group. However, we investigated just four samples of MS patients and healthy subjects. The effect of GA on IL-17, IFN-γ, and IL-10 mRNA expression in CD4^+^ T cells needs further investigation.

Taken together, these results suggest that GA may act not only through APCs but also directly on T cells. Furthermore, according to some authors, levels of IL-17 (in PBMCs culture supernatant) and IFN-γ (in sera) might be predictive biomarkers of clinical response to treatment with GA. Thus, Valenzuela et al. [27] reported that treatment with GA resulted in a tendency toward decreased levels of IL-17 and IFN-γ and an increase of TGF and IL-10 at 3 and 6 months into treatment in clinical responders relative to non-responders. However, this needs to be confirmed by independent studies.

These data are in line with data from other groups who showed that GA inhibits Th1- and Th17-signaling pathways, suppresses the plasma level of IL-17 and induce IL-10 producing regulatory T cells in mice with EAE [28–30].

In particular, it was shown that GA treatment suppresses mRNA expression of Th1-polarization factor STAT-4 and Th17-differentiation factor RORγt in the brain tissue of mice with EAE [27]. In the other study, it was shown the inhibitory effect of GA (5 μg/ml and 10 μg/ml) on STAT-1 and STAT-3 phosphorylation in glial cells co-cultured with anti-CD3 and anti-CD28 stimulated lymphocytes of mice. It also has been shown the inhibitory effect of GA on IFN-γ- and LPS-stimulated phosphorylations of STAT-1 and STAT-3 in glial cells. The same results were obtained in the study of the effect of GA on STAT-1 and STAT-3 activation in LPS- or IFN-γ-stimulated PBMCs. In subsequent experiments, it was shown that the induction of STAT-regulating molecules (suppressors of cytokine signaling 1 and 3 (SOCS-1 and SOCS-3)) could mediate the inhibitory effect of GA on STAT-1 and STAT-3 activation [30]. Besides the RORγt, STAT-3 is also critical for Th17 differentiation. Cytokines signaling (including IL-1, IL-6, IL-21, IL-23, and TGF-β) activate STAT-3, which directly regulates the *IL17* gene and is necessary for the expression of various transcription factors involved in Th17 differentiation [31]. In the current study, we have found an inhibitory effect of GA on IL-1β and IL-6 production by DCs–that also could be the reason for the decrease of STAT-3 signaling and subsequent decreasing IL-17 production.

It is important to note that T cells and monocytes obtained from MS patients during relapse had higher levels of activated STAT-3 and lower levels of SOCS-3 in comparison with cells obtained from MS patients during remission [32].

It is important to discuss the concentrations of GA we used in this study. Previously it was reported that the modulatory effects of GA on PBMCs at concentrations more than 200 μg/ml are at least in part due to toxic effects. At concentrations lower than 200 μg/ml, anti-inflammatory effects of GA cannot be explained by unspecific toxicity [21]. In line with these data, we used several GA concentrations– 50 μg/ml, 100 μg/ml and 200 μg/ml.

The highest GA concentration was 200 μg/ml. Such a large amount of GA was used according to literature data–as the maximum GA concentration in several *in vitro* and in vivo studies was 200 μg/ml and higher [21, 33, 34]. In the recent study on the role of GA in B cells as a modulator of Ca2^+^ homeostasis, the GA concentration of 100 μg/ml was used [35].

In the current study, some effects of GA were seen only using the highest concentration, but most results were observed using the 50 μg/ml and 100 μg/ml of GA. At these concentrations (50 μg/ml and 100 μg/ml), GA had a clear inhibitory effect on IL-17 and IFN-γ production by PBMCs and CD4^+^ T cells in both groups. At the same time at a concentration of 50 μg/ml, there was no effect of GA on IL-10 production by CD4^+^ T cells in both groups. In that way, the most important results were seen with 50 μg/ml.

According to some studies, the effect of GA is also observed at concentrations of lower than 50 μg/ml. For instance, at a dose of 20 μg/ml, GA induced a lymphoproliferative response in PBMCs of MS patients (in 8 of 12 patients) [36]. In the study by Karandikar et al. there was a robust proliferative response to GA (30 μg/ml) of CD4^+^/CD8^–^ and CD8^+^/CD4^–^ T cells of both healthy controls and MS patients. This study also revealed that untreated MS patients have a deficient CD8^+^ T cell response to GA compared to healthy individuals. Treatment with GA results in the restoration of these CD8^+^ responses to levels observed in healthy donors [37].

Another study revealed a significant decrease of IFN-γ and an increase of IL-4, IL-5, and IL-10 in the supernatants of GA-stimulated (20 μg/ml) PBMCs of GA-treated MS patients in the first year of treatment [38]. However, we have not found studies evaluating the impact of GA on IL-17 production by PBMCs and CD4^+^ T cells–as it had been researched in current study.

It is important to compare these concentrations with GA concentration in the body. However, it is rather difficult to assess GA concentrations in vivo because GA undergoes rapid degradation after subcutaneous injection [39]. According to Schmidt et al. [21], systemic GA concentrations of this magnitude are highly unlikely to occur in vivo. Glatiramer acetate is usually prescribed at the dose of 20 mg daily or 40 mg three times a week [26, 40]. The maximum observed concentration of GA in plasma was 0.07–0.6 μg/ml after subcutaneous injection of a single, supratherapeutic dose of 60 mg, to healthy volunteers [41]. That is more than 80 times lower than the minimal concentration used in this work. On the other hand, it is possible for GA or its derivatives to accumulate in some compartments, e.g. in lymphoid tissues. In this case, it is possible to suggest that concentrations of GA used in this study may be comparable with GA concentration in regional lymph nodes.

We also studied the influence of GA on DC-mediated Th17-immune response. First, we studied the effect of GA on IL-6 and IL-1β production by DCs. Previous studies have shown that IL-23 promotes differentiation and proliferation of Th17 cells [42]. At the same time, TGF-β, IL-6 and IL-1β are also necessary for the differentiation of Th17 cells [43]. In the absence of IL-6 or IL-1β, the TGF-β signaling pathway induces the differentiation of regulatory T cells, which prevent autoimmune diseases by inducing anti-inflammatory cytokines [42]. It was shown that Th17 cells differentiated with TGF-β 1 and IL-6 do not induce EAE when transferred to naïve recipients [16].

We found an inhibitory effect of generic GA on IL-1β production in both groups. This data correspond with data from other authors who have shown that GA treatment enhanced IL-1 receptor antagonist blood levels in both EAE mice and MS patients and suppressed IL-1β production by LPS-activated human monocytes *in vitro* [44, 45]. We also found that GA suppressed IL-6 production by DCs in MS patients and healthy subjects. These data are in line with data by Begum-Haque who reported the inhibitory effect of GA on IL-6 mRNA expression in brain in EAE mice [28]. However, it is possible that inhibitory effect of GA on IL-6 production in MS patients in our study could be associated with non-specific toxicity of high concentration of GA (200 μg/ml) on DCs. Nevertheless, the influence of GA on IL-6 is not fully understood. According to Dabbert et al. [46], GA-specific T cell clones secreted IL-6. Furthermore, IL-6 can also promote Th2 differentiation by inducing IL-4 production and blocking IFN-γ signaling [47, 48]. In this regard, therapy with GA could be interesting to study the dual pro- and anti-inflammatory role of IL-6 [49].

In line with inhibition of IL-6 and IL-1β production, generic GA diminished the ability of DCs to induce IL-17 production by autologous CD4^+^ T cells in both study groups. However, effect of GA on IL-17 production in this system was only observed when DCs had been treated with GA at 200 μg/ml, whereas in experiments with PBMCs and purified T cells, GA was effective at lower concentrations (100 and 50 μg/ml). Taken together, these data suggest that GA can inhibit on Th17-immune response and that this inhibitory effect is preferentially exercised by direct influence of GA on T cells.

Finally, in this study we demonstrate the comparable effects of generic and original GA on pro-inflammatory cytokine production *in vitro*. The cost of DMTs including GA remains high, highlighting the potential value of generic therapies for MS. Expiration of patents for the original GA allows to create generics of GA with the expected cost savings for payers and patients. Timexon^®^ is generic GA manufactured in Russia. In a clinical trial (ClinicalTrials.gov Identifier: NCT02753088), comparable efficacy and safety of Timexon^®^ and original GA Copaxone^®^ has been shown [19]. However, GA is a complex mixture of synthetic polypeptides with a range of molecular weights and sequences, manufactured by copolymerization of four amino acids (L-glutamine, L-lysine, L-alanine, L-tyrosine) at a specific molar ratio [50]. Therefore, additional studies to demonstrate equivalence are needed. In this study, we provide a model for comparisons of original and generic GA based on the ability of GA to modulate T cell cytokine production.

## Supporting information

S1 Table PCR primers used in the study. (TIF)

S1 Fig The phenotype of human immature monocyte-derived DCs. The phenotype of dendritic cells (DCs) was determined by multi-color flow cytometry. DCs (8 х 104 per 50 μl) were stained with an fluorescein isothiocyanate (FITC)-labeled monoclonal antibody (mAb) to CD11c (Miltenyi Biotec, Germany) in combination with phycoerythrin (PE)-labeled mAb to HLA-DR (Miltenyi Biotec) (A) or PE-labeled mAb to CD14 (Beckman Coulter, USA) (B) or PE-labeled mAb to CD19 (Beckman Coulter) and PC5-labeled mAb to CD3 (Beckman Coulter) (C and D), after which the samples were resuspended in 200 μl of phosphate-buffered saline (PBS, pH7.3) and analyzed with a 4channel FACSCalibur flow cytometer using CellQuest software (Becton Dickinson, USA). The antibodies of the same isotypes and labels, but without antigenic targets in human cells (isotype control) were added into the controlsamples (E, F, G).
(PDF)

S1 Data Experimental data. (XLSX)
