# Supplementary figures and images for: The influence of glatiramer acetate on Th17-immune response in multiple sclerosis
Source: PLoS One. 2020 Oct 30;15(10):e0240305. doi: 10.1371/journal.pone.0240305 (PMC7599084; doi:10.1371/journal.pone.0240305)

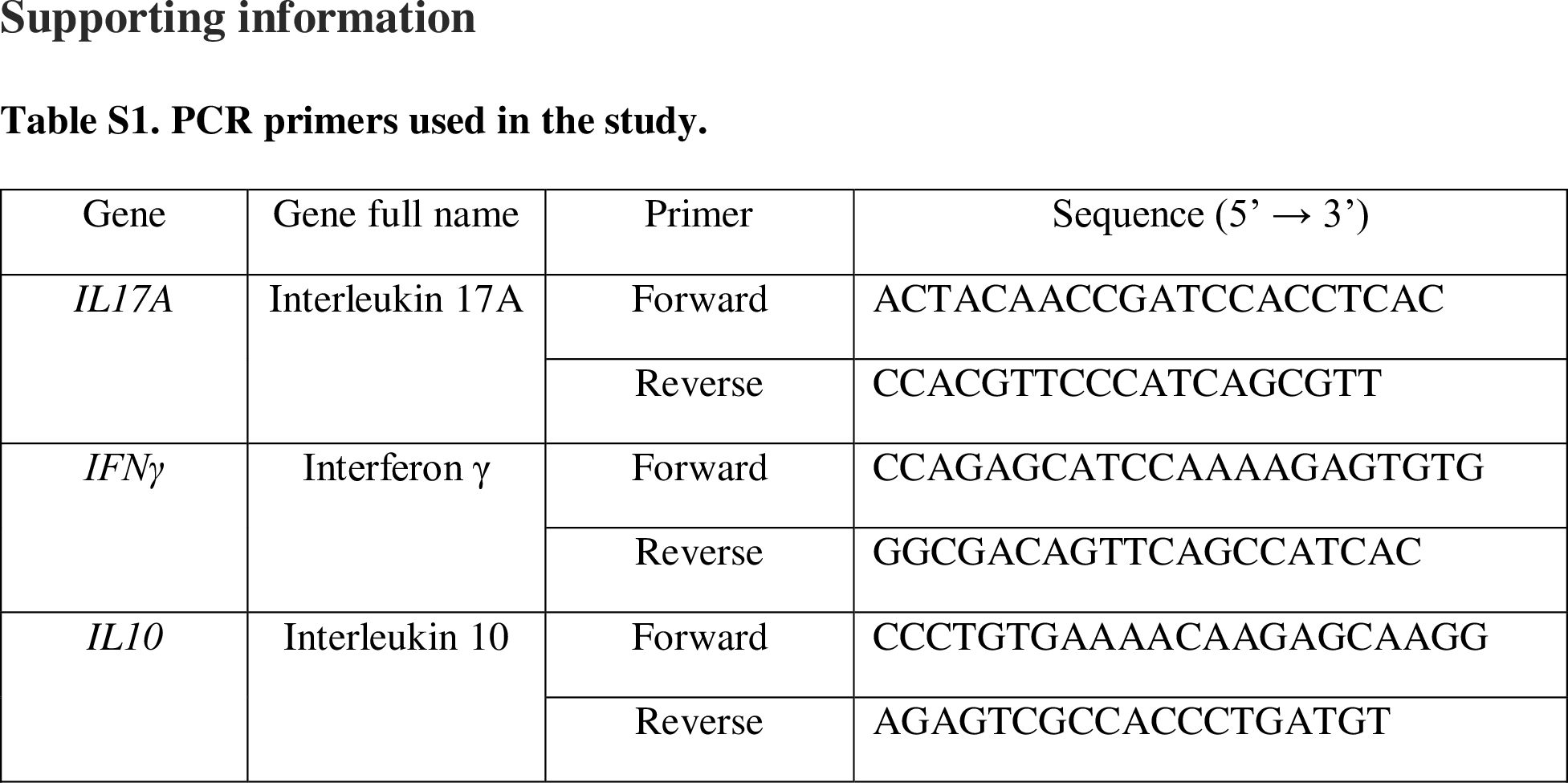

Supplement: S1 Table — (TIF) [file pone.0240305.s001.tif]
