# Supplementary material for: The influence of glatiramer acetate on Th17-immune response in multiple sclerosis
Source: PLoS One. 2020 Oct 30;15(10):e0240305. doi: 10.1371/journal.pone.0240305 (PMC7599084; doi:10.1371/journal.pone.0240305)
Supplement: S1 Fig — The phenotype of dendritic cells (DCs) was determined by multi-color flow cytometry. DCs (8 х 104 per 50 μl) were stained with an fluorescein isothiocyanate (FITC)-labeled monoclonal antibody (mAb) to CD11c (Miltenyi Biotec, Germany) in combination with phycoerythrin (PE)-labeled mAb to HLA-DR (Miltenyi Biotec) (A) or PE-labeled mAb to CD14 (Beckman Coulter, USA) (B) or PE-labeled mAb to CD19 (Beckman Coulter) and PC5-labeled mAb to CD3 (Beckman Coulter) (C and D), after which the samples were resuspended in 200 μl of phosphate-buffered saline (PBS, pH7.3) and analyzed with a 4channel FACSCalibur flow cytometer using CellQuest software (Becton Dickinson, USA). The antibodies of the same isotypes and labels, but without antigenic targets in human cells (isotype control) were added into the controlsamples (E, F, G). (PDF) [file pone.0240305.s002.pdf]

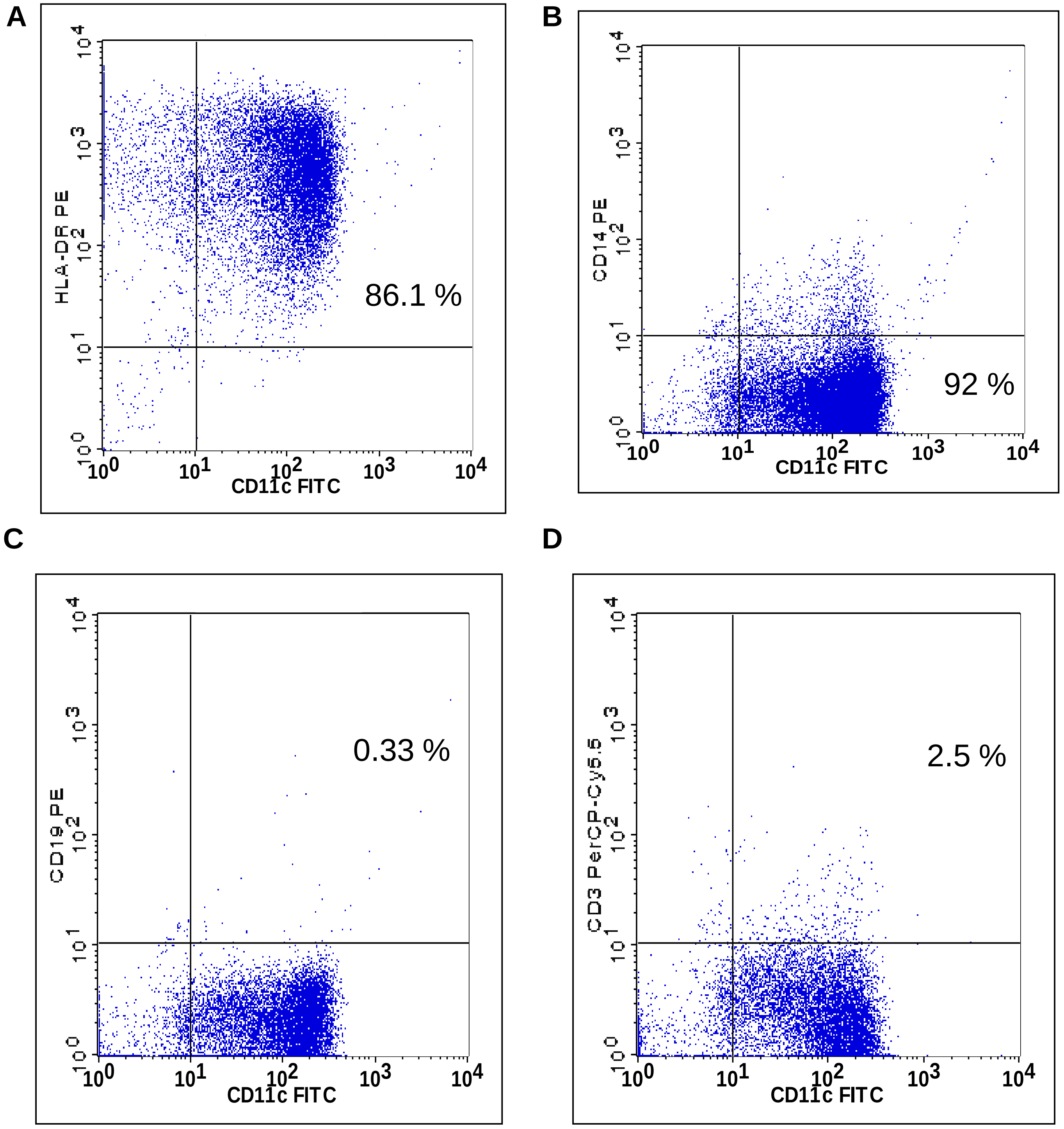
A
HLA-DR PE
CD11c FITC
86.1 %
B
CD14 PE
CD11c FITC
92 %
C
CD19 PE
CD11c FITC
0.33 %
D
CD3 PerCP-Cy5.5
CD11c FITC
2.5 %

E

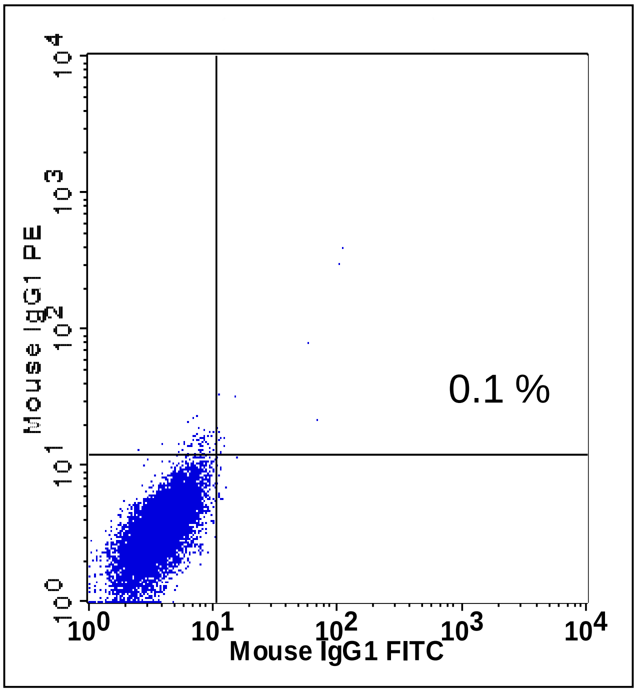

F

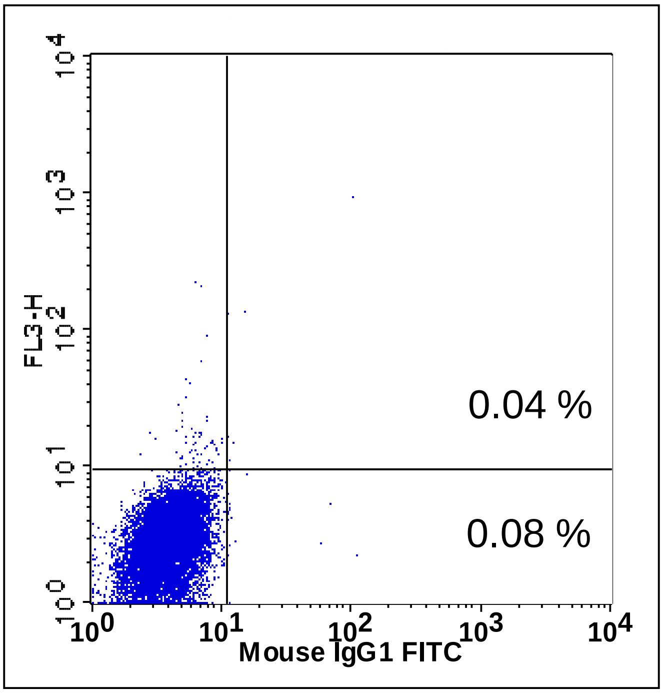

G

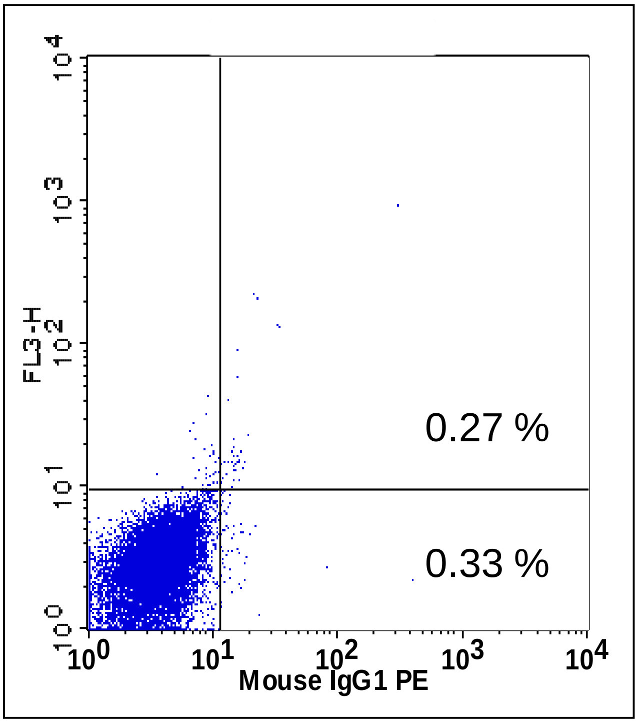

Fig S1. The phenotype of human immature monocyte-derived DCs.

The phenotype of dendritic cells (DCs) was determined by multi-color flow cytometry. DCs (8 x 104 per 50 µl) were stained with an fluorescein isothiocyanate (FITC)-labeled monoclonal antibody (mAb) to CD11c (Miltenyi Biotec, Germany) in combination with phycoerythrin (PE)-labeled mAb to HLA-DR (Miltenyi Biotec) (A) or PE-labeled mAb to CD14 (Beckman Coulter, USA) (B) or PE-labeled mAb to CD19 (Beckman Coulter) and PC5-labeled mAb to CD3 (Beckman Coulter) (C and D), after which the samples were resuspended in 200 µl of phosphate-buffered saline (PBS, pH7.3) and analyzed with a 4-channel FACSCalibur flow cytometer using CellQuest software (Becton Dickinson, USA). The antibodies of the same isotypes and labels, but without antigenic targets in human cells (isotype control) were added into the control samples (E, F, G).
